# Roles of the Oxidative Stress and ADMA in the Development of Deep Venous Thrombosis

**DOI:** 10.1155/2014/703128

**Published:** 2014-04-13

**Authors:** Meral Ekim, M. Ramazan Sekeroglu, Ragıp Balahoroglu, Halil Ozkol, Hasan Ekim

**Affiliations:** ^1^School of Health, Bozok University, 66200 Yozgat, Turkey; ^2^Department of Biochemistry, Yuzuncu Yil University Medical Faculty, 65080Van, Turkey; ^3^Department of Medical Biology, Yuzuncu Yil University Medical Faculty, 65080 Van, Turkey; ^4^Department of Cardiovascular Surgery, Bozok University Medical Faculty, 66200 Yozgat, Turkey

## Abstract

Venous thromboembolism has multifactorial origin and occurs in the context of complex interactions between environmental and genetic predisposing factors. Oxidative stress plays an important role in the physiopathology of venous thrombosis. Current study examined the role of oxidative stress and asymmetric dimethylarginine in the development of DVT with the parameters such as serum malondialdehyde (MDA), glutathione peroxidase (GSH-Px), catalase, ADMA, homocysteine, folic acid, vitamin B_6_, and vitamin B_12_ levels. Serum MDA levels were found significantly (*P* < 0.005) high in patients with DVT compared with control group. Additionally, serum B_6_ levels were found significantly (*P* < 0.009) low in patients with DVT compared with healthy volunteers. There were no significant differences between the groups in terms of the other parameters (*P* > 0.05). This study showed that patients with DVT have increased oxidative stress compared with the healthy volunteers whereas there was no significant difference between the groups in terms of serum ADMA levels. Thus serum ADMA levels seemed to be not related with development of DVT.

## 1. Introduction

Venous thromboembolism (VTE) is a complex vascular disease that may result from many etiologic factors. The first and more common clinical form of VTE is deep venous thrombosis (DVT). Coexistence pulmonary embolism with DVT comprises the second, potentially lethal clinical form of VTE and carries high mortality risk [1]. Thrombus formation and propagation depend on the presence of abnormalities of blood flow, blood vessel wall, and blood clotting components, known collectively as Virchow triad [2]. The processes that trigger venous thrombosis are not obvious. However, it is clear that the mechanisms initiating the venous thrombosis are very different from those initiating the arterial thrombosis [3]. Endothelial imbalance may possibly play an important role [4]. Vascular endothelial surface normally creates a nonthrombogenic structure. When endothelial injury occurs related to factors such as anoxia, mechanical stress, free radicals, cytokines, and thrombin, it may lead to platelet activation and coagulation [5].

Oxidative stress is defined as an imbalance between the oxidant and antioxidant systems and results from either overproduction of free oxygen radicals or insufficiency of antioxidant mechanisms. Recent studies have shown that increased free oxygen radicals and lipid peroxidation play important role in the pathogenesis of many diseases. Serum MDA level is a remarkable indicator for lipid peroxidation and widely accepted as a marker of free radical activity [6].

Nitric oxide (NO) is synthesized in the endothelial cells from the conversion of L-arginine to L-citrulline through the tightly regulated activity of endogenous nitric oxide synthase (NOS) [7]. It modulates vasomotor tone, inhibits platelet aggregation, and smooths muscle cell (SMC) proliferation [8]. Oxidative stress decreases the half-life of NO [9]. NO may play a role in hemostatic plug formation and in thrombotic process with the effects on blood platelets. However, in vivo studies on the role of NO in thrombotic process are not well defined. Most of the previous studies focused on the antithrombogenic properties of NO in arterioles, rather than in venules [10]. A novel risk factor for development of endothelial dysfunction (ED) and cardiovascular disease is asymmetric dimethylarginine (ADMA), a competitive endogenous inhibitor of nitric oxide synthase (NOS) [11]. High levels of serum ADMA levels are associated with vascular disease [12].

In this study, we investigate the role of oxidative stress and asymmetric dimethylarginine as a novel marker of cardiovascular disease in the development of DVT.

## 2. Material and Methods

Our study was conducted in accordance with the regulations of the local ethics committee and the declaration of Helsinki.

Thirty-five consecutive patients with symptomatic lower extremity proximal unprovoked DVT admitted to Yuzuncu Yil University Hospital were included in this prospective study. Their diagnoses were made in emergency service and policlinics. We excluded patients with cardiovascular risk factors such as diabetes mellitus, hyperlipidemia, hypertension, and obesity. Besides, patients were excluded if they were suspected to have pulmonary embolism, if they were pregnant, if they used oral contraceptives, and if they were cigarette smokers or alcoholics. None of them had a past and familial history. Our study protocol was approved by our local ethic committee, and written informed consents of all participants were obtained before the conduction of the study.

In all patients, the diagnosis of DVT was established by physical examination, Wells scoring system and serum D-dimer levels and confirmed by Doppler ultrasonography. Patients with low Wells score and negative D-dimer test (<500 ng/mL) were excluded from the study. Of 35 patients, 21 were female (mean age 40.44 years) and 14 were male (mean age 50.41 years) ranging in age from 20 to 64 years. The control group consisted of 34 apparently healthy volunteers (20 female, mean age 40.11 years, and 14 male, mean age 52.26 years), ranging in age from 22 to 73 years. Obesity was considered for persons with BMI > 30 kg/m^2^. Dyslipidemia was considered for persons with total cholesterol > 2.5 g/L and/or triglycerides > 2 g/L. The two groups were well matched according to age, body mass index (BMI), and gender. The Wells score was equal to or higher than 2 in all patients.

## 3. Blood Chemistry 

A total of 10 mL of fasting blood samples were collected into the test tubes that did not contain any anticoagulants. Blood samples were centrifuged at 5000 rpm for 5 minutes. The serum samples were stored at −70°C until analysis. On examination of the samples, serum malondialdehyde (MDA), asymmetric dimethylarginine (ADMA), catalase (CAT), glutathione peroxidase (GSH-Px), homocysteine, vitamin B_6_, Vitamin B_12,_ and folic acid levels were measured. Measurements were performed within 3 months.

## 4. Measurements and Calculations

Serum concentration of ADMA, MDA, homocysteine (total), and vitamin B_6_ was measured by high performance liquid chromatography (HPLC) (Agilent 1100 series, Germany) using precolumn derivatization with o-phthaldialdehyde (OPA). Folic acid and vitamin B_12_ were determined with competition chemiluminescence enzyme immunoassay method (Immulite 2000, USA).

Serum catalase activity was measured according to the colorimetric method of Goth [13]. Serum was incubated with hydrogen peroxide (H_2_O_2_), and the reaction was stopped by adding ammonium molybdate. The absorbance of the yellow complex formed by molybdate and H_2_O_2_ was measured at 405 nm spectrophotometrically. Serum CAT activity was expressed as Ul^−1^.

Serum GSH-Px activity was determined using the method described by Pleban et al. [14]. In the presence of glutathione reductase and NADPH, the oxidized glutathione is immediately converted to the reduced form with a concomitant oxidation of NADPH to NADP^+^. The decrease in absorbance at 340 nm is measured. Serum GSH-Px activity was expressed as Ul^−1^.

## 5. Statistical Analysis

All parametric values were expressed as the mean ± standard deviation. The difference between groups was compared by independent-samples* t*-test. Pearson correlation analyses were used in the statistical evaluation of the relationship between parameters.

## 6. Results 

There were no significant differences between both groups regarding age, gender, and BMI (Table 1). Serum MDA levels were noted significantly (*P* < 0.005) high in patients with DVT compared with in control group. Additionally, serum vitamin B_6_ levels were found significantly (*P* < 0.009) low in patients with DVT compared with healthy volunteers. There were no significant differences between the groups regarding other parameters (Table 2).

No correlations were observed among parameters related to control group. On the other hand, correlations were observed among some parameters related to study group (Table 3). There was statistically significant positive correlation between MDA and vitamin B_12_ and ADMA and folic acid (*P* < 0.01).

## 7. Discussion

DVT is believed to be a multifactorial disease and occurs in the context of complex interactions between environmental and genetic predisposing factors. In addition, DVT is characterized by small flow velocity and low shear rate, but large thrombotic mass is a good model to investigate the role of oxidative stress [15]. In our study, we found higher MDA (lipid peroxidation marker) levels in DVT patients compared to the healthy controls. These increased levels of MDA indicate lipid peroxidation in patients with DVT. The only study we could find in the literature on this subject is the study of Re et al. [16]. They found higher MDA and hydroxynonenal (HNE) and myeloperoxidase (MPO) levels in DVT patients compared to the healthy controls. The most important difference of the current study from the previous studies is the investigation of the contribution of antioxidant enzymes glutathione peroxidase and catalase.

Under normal physiological conditions, the antioxidants are responsible for cellular protection against oxidative stress, namely, by the free radical scavenger enzyme superoxide dismutase (SOD), catalase, and glutathione peroxidase (GSH-Px) [17], thereby maintaining the vasorelaxant and antithrombotic effects of nitric oxide (NO) in the vasculature [18]. These scavengers are strategically compartmentalized in subcellular organelles within the cell to provide maximum protection [19]. The glutathione peroxidases (GSH-Px) are a family of enzymes which reduce oxidized lipids to their nontoxic metabolites and may thereby decrease vascular injury. Previous studies have suggested that low levels of both GSH-Px1 and GSH-Px3 are associated with the development of vascular disease. GSH-Px3 activities may have declined as a result of the disease process rather than being its cause [20]. In addition to its antioxidant properties, GSH-Px also enhances the bioavailability of NO by catalyzing its liberation from s-nitrosothiols and reducing lipid peroxides, and glutathione peroxidase potentiates the inhibition of platelet function by NO by reducing LOOH [21]. Oxidative reactions involving NO in plasma can attenuate its effect on platelet function and, subsequently, induce thrombosis [22]. A deficiency in plasma GSH-Px leads to an increase in plasma peroxides that can lead to inactivation of NO (via peroxyl-mediated formation of [lipid] peroxynitrites) [23]. It has demonstrated that an innate GSHPx-3 (a member of the selenocysteine-containing GSH-Px) deficiency impairs bioavailable NO and leads to platelet hyperreactivity and an increased risk of arterial thrombosis. In addition, Vadseth and colleagues [24] demonstrated that prothrombotic state is induced by posttranslational modification of fibrinogen by reactive nitrogen species.

There is evidence suggesting a role for oxidative stress in the pathobiology of venous thrombosis [16]. We have found no studies related to association of GSH-Px and catalase enzyme activities with venous thrombosis. In this study, GSH-Px and catalase activities were measured as an indicator of antioxidant enzyme capacity, and no significant difference of antioxidant enzyme capacity (GSH-Px and CAT) was detectable between both groups (Table 2). We conclude that antioxidant enzyme capacity is not altered by DVT. Thus, these enzymes do not play a role in thrombosis under low flow conditions. Though GSH-Px deficiency increases the risk of arterial thrombosis [23], its deficiency may not seem to represent a risk factor for a prothrombotic state in the development of venous thrombosis.

Broeders et al. [10] showed that NO plays a more important role in inhibiting thromboembolism in venules than in arterioles. Chronically elevated ADMA blood levels may contribute to progression of vascular disease via endothelial damage. This effect seems to involve more than just reduced NO availability secondary to NOS inhibition [25]. In addition to inhibiting production of NO, ADMA may also promote the uncoupling of eNOS, directly contributing to increased oxidative stress [26]. ADMA is increased in chronic thromboembolic pulmonary hypertension (CTEPH), a condition frequently associated with DVT [27]. Also, plasma ADMA levels were found to be significantly higher in patients with idiopathic pulmonary arterial hypertension (IPAH). ADMA may be a relevant factor in the pathogenesis of IPAH [28].

Enhanced endogenous ADMA production may, therefore, contribute to the establishment of a prothrombotic state. Although increased blood concentrations of ADMA can be found in patients with cardiovascular risk factors [25], in this study, there was a small increase in the ADMA level in DVT patients compared to the control group, and this was not statistically significant. The only study we could find in the literature related to this issue is the study of Haider et al. [29]. Consistent with our finding, they also concluded that circulating ADMA concentrations are not altered by venous thromboembolism [29]. In a recent animal study, it might be worthwhile to mention that even long term infusion of ADMA (in a preclinical model) did at least not affect peripheral numbers of erythrocytes, leukocytes, and thrombocytes [30].

Homocysteine is a sulfhydryl-containing amino acid formed during the metabolism of methionine [31]. Elevation of plasma homocysteine level is a risk factor for cardiovascular disease, stroke, and venous thromboembolism [29]. Mild hyperhomocysteinemia is caused by genetic factors or acquired conditions, such as inadequate intake of those vitamins that are involved in homocysteine metabolism (folic acid, cobalamin, and vitamin B_6_), renal insufficiency, and some medications. However, the association between hyperhomocysteinemia and venous thrombosis remains controversial [32]. The pathophysiological mechanisms leading to vascular damage in hyperhomocysteinemia are multifactorial. Indeed there is accumulating evidence from animal and human studies that ADMA, endothelial dysfunction, and homocysteine might be closely interrelated [33]. Although, in the current study, there was a small increase in the homocysteine level in DVT patients compared to the control, and this was not statistically significant. Furthermore, in this study, there was no statistically significant difference in the levels of vitamin B_12_ and folic acid between the groups. But, vitamin B_6_ levels were significantly lower in DVT patients than those in healthy controls. Hron and colleagues [34] noticed that low vitamin B_6_ level is a risk factor of recurrent venous thromboembolism. It was found that low levels of pyridoxal-5-phosphat, the coenzyme form vitamin B_6_ < 21, 7 nmol/L gave a twofold higher thrombotic risk [35]. In addition, Zhou et al. [36] reported that there may be a homocysteine-independent role for B-group vitamins in venous thrombosis development. Our finding supports opinion that it may be possibly a homocysteine-independent relationship between low vitamin B_6_ level and venous thrombosis.

Finally, our data showed that patients with DVT have increased oxidative stress compared with the healthy volunteers. However, there was no significant difference between the groups in terms of serum ADMA levels in our study. Thus serum ADMA levels may not be a predictive risk factor for development of DVT.

The single institutional setting and a small sample of patients are the main limitations of our study, and thus the conclusions should be supported by future research in the field.

## Figures and Tables

**Table 1:** Main clinical characteristicsof study subjects.

	DVT patients (*n* = 35)	Controls (*n* = 34)
Male/female (*n*)	14/21	14/20
Age, years	45.42 ± 13.82	46.18 ± 10.39
Body mass index (kg/m^2^)	25.98 ± 3.35	25.50 ± 4.00

**Table 2:** Serum parameters in both groups.

Parameters	Control group	DVT patients
GSH-Px (U/L)	467.49 ± 88.47	461.24 ± 87.88
Catalase (kU/L)	354.38 ± 113.58	347.24 ± 106.48
MDA (*μ*mol/L)	0.09 ± 0.05	**0.13 **± 0.06*
Hcy (*μ*mol/mL)	12.31 ± 4.51	15.22 ± 9.26
ADMA (*μ*mol/L)	0.37 ± 0.19	0.51 ± 0.38
Vit B_6_ (*μ*g/L)	6.61 ± 4.91	**3.96 **± 3.00*
Vit B_12_ (pg/mL)	297.09 ± 161.07	257.62 ± 163.12
Folic Acid (ng/mL)	7.73 ± 9.58	4.86 ± 4.00

Data were expressed as mean (SD) ± standard deviation.

*P < 0.01 (compared to the control group).

GSH-Px: glutathione peroxidase, MDA: malondialdehyde, Hcy: homocysteine, ADMA: asymmetric dimethylarginine, Vit B_6_: vitamin B_6_,  and Vit B_12_: vitamin B_12_.

**Table 3:** The relationship between measuring parameters in DVT patients with Pearson correlation.

	GSH-Px	Catalase	MDA	Hcy	ADMA	Vit B_6_	Vit B_12_
Catalase	−0.05						
MDA	0.01	0.18					
Hcy	−0.1	0.29	−0.2				
ADMA	0.23	−0.14	0.21	−0.21			
Vit B_6_	0.12	0.3	−0.01	0.03	−0.23		
Vit B_12_	0.13	0.19	0.43*	−0.23	0.11	0.27	
Folic acid	0.21	−0.11	0.27	−0.23	0.58*	0.04	−0.01

*P < 0.01.

MDA: malondialdehyde, Hcy: homocysteine, ADMA: asymmetric dimethylarginine, Vit B_6_: vitamin B_6_, and Vit B_12_: vitamin B_12_.
